# Female sling: handmade adjustable threads

**DOI:** 10.1590/S1677-5538.IBJU.2019.0432

**Published:** 2020-11-18

**Authors:** Luis Gustavo Morato de Toledo, Rafael Ribeiro Zanotti, André Costa Matos

**Affiliations:** 1 Santa Casa de São Paulo Faculdade de Ciências Médicas Departamento de Cirurgia SP Brasil Seção de Urologia do Departamento de Cirurgia da Faculdade de Ciências Médicas da Santa Casa de São Paulo, SP, Brasil; 2 Hospital São Rafael Departamento de Urologia Rede D'orBA Brasil Departamento de Urologia, Hospital São Rafael - Rede D'or, BA, Brasil

## Abstract

**Introduction:**

The optimal fit of a sling mesh for the treatment of stress urinary incontinence (SUI) in patients with associated pelvic organ prolapse (POP), or other predictors of sling failure, such as fixed urethra and previous incontinence surgery, can be challenging. The ideal point of tension may not be accurate, when the tape is too loose, incontinence persists. On the other hand, when the tape is too tight, urinary obstruction is produced. For these patients, adjustable slings are desirable.

**Objective:**

The purpose of this article and accompanying video is to show that synthetic middle urethral slings can be adjustable if necessary.

**Materials and Methods:**

In our report and accompanying video, we demonstrate and explain the technique to make handmade adjustable sling threads. The selected tape was an Obtryx Halo™, but the technique can be employed to any synthetic middle urethral sling, retropubic or transobturator. We also show the adjustments at the post-operative days. That patient underwent colpocleisis and transobturator sling. On first postoperative day, before removal of the catheter, bladder is filled with 200 to 300mL of saline solution. After catheter removal, cough stress test is performed and if leakage occurs, the sling is tightened (Figure-1). Complete emptying must be certified by post-voiding catheterization, especially if tightening has been performed. In case of over tensioning, the suture for loosening may be used (Figure-2). The patient is discharged from the hospital after optimal adjustment of the sling tension has been obtained. Early outpatient returns are scheduled for stress and voiding testing, and so, new adjustments if necessary. Adjustment sutures are removed 3 to 7 days after the last adjustment. Sling adjustment is painful, local anesthesia may be applied to the sling path through the skin. The patient is maintained on antibiotic prophylaxis with cephalosporin until the adjustable sutures are removed.

**Results:**

The patient was maintained catheterized for 24 hours and discharged at first post-operative day, when we did the first tightening adjustment. Another adjustment was necessary at outpatient follow-up on sixth post-operative day. She was dry on tenth post-operative day, without urinary residual and with normal uroflowmetry parameters; so, we cut out the threads. The patient had no recurrence of genital prolapse and no leakage, she was fully satisfied one year after surgery.

**Conclusions:**

Synthetic middle urethral slings can become adjustable for the first 7 to 10 post-operative days. Late displacement of the sling can be too painful and is not recommended. The adjustment will benefit a restrict number of patients with poor prognosis factors (urethral hypomobility, advanced stage POP, previous SUI surgery, detrusor underactivity), when the successful sling tensioning range is narrow (Figure-3). It can prevent immediate failure for this group of patients. This procedure represents a safe, effective, and low-cost technique for SUI treatment, and does not increase the risk of infection nor erosion or other negative effect.

**Figure 1:**
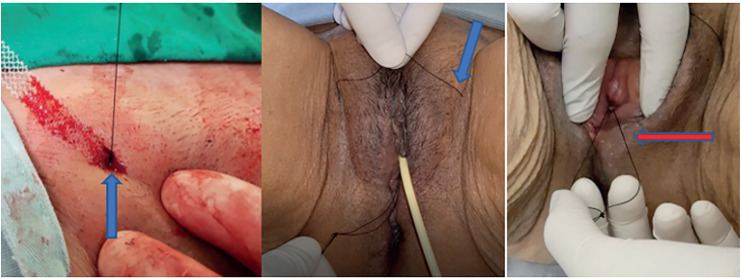
Blue arrow: retropubic (left) and transobturator (middle) threads for tightening, red arrow: thread for loosening.

**Figure 2:**
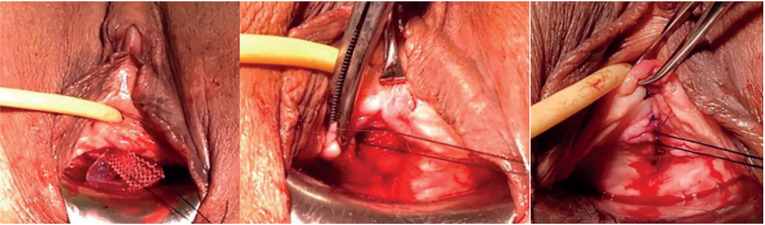
Central thread for loosening. Positioned 1-2cm laterally to the urethral midline, so the mesh does not come out through the vaginal suture if loosening is necessary.

**Figure 3:**
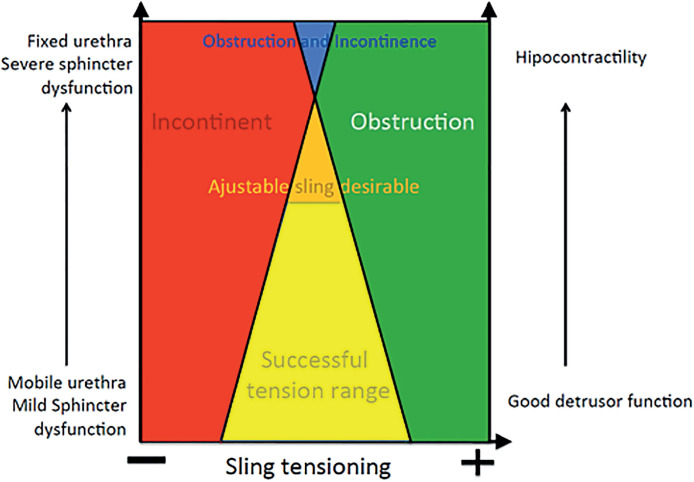
Diagram showing different patient presentations, at the bottom we have patients with good urethral mobility, mild sphincter dysfunction and good detrusor function; therefore, a wide range of successful sling tensioning. As you go up towards the patients tending to severe sphincter dysfunction, detrusor underactivity and fixed urethra, the successful range gets progressively narrower, so in this occasion the adjustable sling is desirable.
